# The role of negative pressure wound therapy—From the perspective of drainage fluid composition

**DOI:** 10.1016/j.sopen.2026.01.003

**Published:** 2026-01-19

**Authors:** Muhaimaiti Abudurezhake, Yifei Huang, Hailong Wang, Gulinuer Aili, Yamei Xu, Yajun Tian, Zhanjun Ma

**Affiliations:** a The Fourth Clinical Medical College of Xinjiang Medical University, China; b Affiliated Hospital of Traditional Chinese Medicine of Xinjiang Medical University, Urumqi, 830000, China; c Wensu County People's Hospital of Xinjiang, Wensu, 843100, China; d Xinjiang Uygur Autonomous Region Institute of Traditional Chinese Medicine, Urumqi, 830000, China

**Keywords:** Negative pressure wound therapy, Drainage liquid, Growth factors, Progenitor cells, Wound healing

## Abstract

Negative pressure wound therapy (NPWT) accelerates wound healing processes by promoting angiogenesis and vascularization. However, the molecular mechanisms and biological effects underpinning these processes remain unclear, while drainage fluids (and associated components) extracted by negative pressure suction are rarely investigated. This study investigated these components and explored their relationship with wound healing. To this end, a diabetic wound rat model was established, and wound exudate was collected using negative pressure wound therapy (NPWT) equipment. Platelet-derived growth factor (PDFG-BB), transforming growth factor-β (TGF-β1), epidermal growth factor (EGF), vascular endothelial growth factor-A (VEGF-A), and stromal cell-derived factor-1 (SDF-1) expression levels were investigated using quantitative reverse transcription polymerase chain reaction (RT-qPCR) and western blotting. Circulating endothelial progenitor cells (EPCs), circulating fibrocytes, and mesenchymal stem cells (MSCs) were analyzed by flow cytometry. This study observed that during wound healing, the expression levels of platelet-derived growth factor (PDGF), transforming growth factor-β (TGF-β1), epidermal growth factor (EGF), vascular endothelial growth factor (VEGF), and chemokine-1 (SDF-1) were significantly higher in the experimental group than in the control group. This expression pattern was similar to that observed in endothelial progenitor cells (EPCs), fibroblasts, and mesenchymal stem cells (MSCs). The data from this study indicate that NPWT significantly increases the clearance of drainage fluid and its related components (including growth factors, chemokines, and cells). Also, drainage fluid levels were proportional to wound healing.

## Graphical abstract

**Figure:**
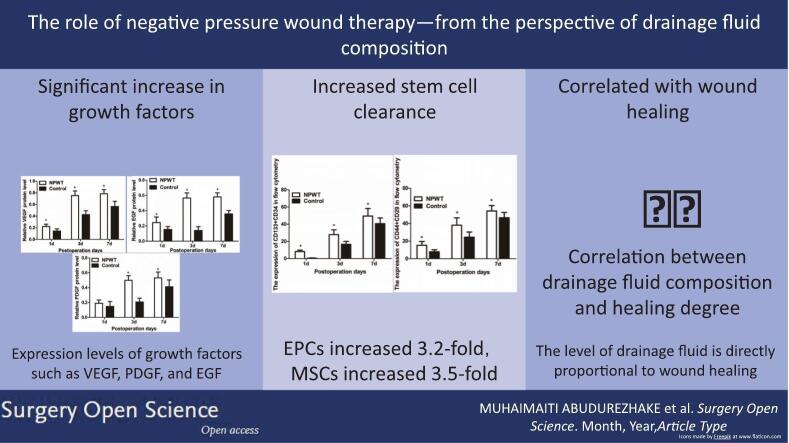

## List of abbreviations

**Abbreviation**
ANOVA: Analysis of Variance
cDNA: Complementary DNA
DMEM: Dulbecco's Modified Eagle Medium
ECM: Extracellular Matrix
EGF: Epidermal Growth Factor
EPCs: Endothelial Progenitor Cells
FBS: Fetal Bovine Serum
FITC: Fluorescein Isothiocyanate
IgG: Immunoglobulin G
MSCs: Mesenchymal Stem Cells
NIH: National Institutes of Health
NPWT: Negative Pressure Wound Therapy
PBS: Phosphate-Buffered Saline
PDGF-BB: Platelet-Derived Growth Factor-BB
PE: Phycoerythrin
PerCP: Peridinin-Chlorophyll-Protein Complex
PI: Propidium Iodide
RT-qPCR: Quantitative Reverse Transcription Polymerase Chain Reaction
SD: Standard Deviation
SDF-1: Stromal Cell-Derived Factor-1
SPF: Specific Pathogen Free
STZ: Streptozotocin
TGF-β1: Transforming Growth Factor-Beta 1
VEGF-A: Vascular Endothelial Growth Factor-A

## Introduction

Negative Pressure Wound Therapy (NPWT) effectively promotes the healing of both acute and chronic wounds [1]. This therapy efficiently drains excessive fluid and debris from the wound, eliminates edema, reduces bacterial colonization, increases local blood perfusion, and promotes granulation tissue formation, thereby accelerating wound closure. In wound management, the macro-deformation process induces wound contraction, characterized by the continuous removal of excess fluid and the maintenance of a warm, moist wound microenvironment [2], [3], [4], [5]. Diabetic wound healing impairment involves multiple pathophysiological factors, including impaired growth factor responses, insufficient angiogenesis, and a chronic inflammatory state [6], [7]. While NPWT has demonstrated clinical value in managing refractory wounds like diabetic foot ulcers [8], the specific mechanisms by which it modulates the wound microenvironment remain unclear. Specifically, does NPWT drainage fluid become enriched or depleted in certain molecules crucial for diabetic wound healing, such as growth factors (e.g., VEGF, PDGF), chemokines (e.g., SDF-1), or reparative precursor cells (e.g., endothelial progenitor cells, mesenchymal stem cells)? Investigating this question will not only deepen our understanding of NPWT's mechanism of action in the context of diabetic wounds but may also provide a scientific basis for optimizing treatment strategies and developing new efficacy monitoring indicators based on drainage fluid composition analysis.

Therefore, this study aims to move beyond the observation of NPWT's macroscopic effects and delve into the molecular and cellular components of its drainage fluid. Using a diabetic rat model with full-thickness skin defects, we will compare the dynamic changes in the levels of key growth factors, chemokines, and circulating progenitor cells in wound drainage fluid under NPWT versus standard gauze dressing treatment. Furthermore, we will explore the correlation between these changes and the wound healing progression. This research aims to provide a scientific foundation for the future refinement of Negative Pressure Wound Therapy (NPWT).

## Materials and methods

### Animals

In total, 30 adult sex-matched Sprague-Dawley rats (Experimental Animal Central Research Laboratory at Wuhan University, Wuhan, China, weighing between 230 and 260 g) were used. Rats were individually caged under specific pathogen-free conditions at a temperature of 24 °C ± 1 °C and relative humidity between 45%–55%. Animals were allowed ad libitum access to water and feed. All experiments were carried out between 09:00 and 17:00 h. Treatment of the animals was carried out in strict accordance with the recommendations described in the Guide for the Care and Use of Laboratory Animals of the National Institutes of Health. This study has been approved by the authors' affiliated institution. All surgical procedures were performed under chloral hydrate anesthesia, and efforts were made to minimize animal suffering.

This study strictly adheres to the “3R”principles of animal experimentation, ensuring animal welfare through the establishment of “humane endpoints.”Humane endpoints are immediately implemented when any of the following conditions are met: (1) The scientific objectives of the experiment have been achieved, rendering further animal experimentation unnecessary; (2) The animal experiences unexpected pain, i.e., pain not caused by the experimental design itself and unforeseeable prior to the experiment's commencement; (3) The animal is experiencing pain that was anticipated in the experiment, but the actual severity of the pain significantly exceeds the pre-experimental estimates. By strictly enforcing these standards, the aim is to minimize the pain and distress that animals may experience during the experiment.

### Wound protocol and animal groups

A total of 30 rats were fasted overnight and then administered a single intraperitoneal injection of freshly dissolved streptozotocin dissolved in 0.1 mmol/L citrate buffer (pH 4.5). A rat diabetes model was successfully established by measuring fasting blood glucose levels. Serum glucose levels were screened 3 days after STZ injection. If fasting blood glucose levels remained above 300 mg/dl for 7 consecutive days, diabetes was diagnosed, and the rats proceeded to the next phase of the experiment. Fasting blood glucose levels were measured three times weekly in all rats. If fasting serum glucose levels exceeded 450 mg/dl, the minimum dose of insulin (0.1–0.2 units/rat) was administered via subcutaneous injection. Throughout the experiment, fasting blood glucose levels were maintained above 300 mg/dl.

In animals, the entire dorsa were clipped and depilated 24 h prior to studies. General intraperitoneal anesthesia was conducted by injection of 350 mg/kg chloral hydrate (7%) 5 min prior to the surgery and no symptoms of peritonitis were found.

The dorsa was disinfected with alcohol patches, and a 3 cm × 3 cm area of skin and panniculus carnosus was removed to provide a full-thickness wound. Rats were then randomly divided into two groups: NPWT (experiment) (*n* = 15) and control groups (n = 15). In the experimental group, the wound was covered with a 3 cm × 3 cm Duoderm dressing (VSD Medical Technology Co., Ltd., Wuhan, Hubei, China) and sealed with a transparent film (VSD Medical Technology Co., Ltd., Wuhan, Hubei, China) using a negative pressure closure device, which was continuously operated at a negative pressure of 125 mmHg. This device did not affect ambulation, diet, or behavior in treated animals. In the control group, wounds were covered with gauze. VSD dressings were replaced twice a week, and dressings were replaced at times when the vacuum assisted closure device malfunctioned. The gauze in the control group was changed three additional times per week.

All experimental animals were first anesthetized by intraperitoneal injection of 350 mg/kg chloral hydrate (7%). Within 48 h post-operation, buprenorphine (0.05 mg/kg) was administered subcutaneously every 12 h for analgesia and to alleviate distress. Subsequently, the animals were euthanized by cervical dislocation on days 1, 3, and 7, and drainage liquids were aseptically collected from each group. In the experimental group, liquids were collected through drain tubes. In the control group, liquids were collected from gauze exudates; specifically, the gauze dressings were immediately placed into 5 mL of ice-cold phosphate-buffered saline after removal and agitated on a shaker at 4 °C for 15 min. The eluent was then centrifuged (1500 ×*g*, 10 min) to remove debris, and the supernatant was collected. The volume of this eluent was recorded and subjected to the same subsequent processing as the NPWT drainage fluid.

### Cell detection and enumeration using flow cytometry

Add pre-cooled phosphate-buffered saline (PBS, 4 °C) to the drainage fluid in the flow cytometry tube. Wash the cells once with PBS in a 6-well plate, then add 1 mL of trypsin (0.25%) to digest the cells. When the cells become round and partially detach, add 2 times the volume of complete culture medium containing 10% fetal bovine serum to terminate digestion, and gently suspend the cells with a pipette. Centrifuge the mixture at 300 *g* for 5 min, discard the supernatant, resuspend the pellet in pre-chilled PBS, and wash twice. Filter through a 40 μm filter, adjust the concentration to 1 × 10^6^ cells/mL, aliquot 100 μL/tube (1 × 10^5^ cells), and add the following fluorescently labeled primary antibodies: anti-rat CD34-FITC (5 μL/tube), anti-rat CD133 + CD29-PE + CD44-PE (10 μL each/tube), and anti-rat CD45-PerCP (5 μL/tube). Mix thoroughly and incubate at 4 °C for 20 min. Add 3 mL PBS, centrifuge at 300 *g* for 5 min, and discard the supernatant. Repeat washing twice, resuspend the pellet in 300 μL PBS, and immediately analyze on the BD FACSCanto II. Set the channels as follows: FITC (Ex = 488 nm, Em = 530/30 nm, FL1), PE (Ex = 488 nm, Em = 585/42 nm, FL2), PerCP (Ex = 488 nm, Em = 675/25 nm, FL3). A isotype control (IgG1-FITC/PF/PerCP) was processed simultaneously to correct for background fluorescence.

### Quantitative RT-PCR (qRT-PCR)

Total RNA was extracted using a RNeasy Mini kit (Qiagen AB, Sollentuna, Sweden) and a thermocycler (Bio-Rad Laboratories, Inc., Hercules, CA, USA). RNA was reverse-transcribed into cDNA using the First Strand cDNA Synthesis kit (Fermentas; Thermo Fisher Scientific, Inc., Waltham, MA, USA) according to manufacturer's protocols. Primer sequences were:

PDGF-BB forward primer, 5′-AAGACCAGGACGGTCATTTACG-3′ and reverse primer, 5′-CTAACCTCACCTGGACCTCTTTC-3′;

TGF-β forward primer, 5′-GTGGCTGAACCAAGGAGACG-3′ and reverse primer, 5′-AGGTGTTGAGCCCTTTCCAG-3′;

EGF forward primer, 5′-GTGATTGCTTTCCTGGGTACG-3′and reverse primer, 5-CCTTCTGGCGTGTCTACTCCTT-3′;

VEGF-A forward primer, 5´-ATCTTCAAGCCGTCCTGTGTG-3′ and reverse primer, 5´-AGGTTTGATCCGCATGATCTG-3′;

SDF-1 forward primer, 5′-GTAAGCCAGTCAGCCTGAGCTAC-3′ and reverse primer, 5′-GGATCCACTTTAATTTCGGGTC-3′; and

GAPDH forward primer, 5′-CGCTAACATCAAATGGGGTG-3′ and reverse primer, 5′-TTGCTGACAATCTTGAGGGAG-3′.

RT-PCR was performed using a SYBR qPCR mix (2×; Toyobo Co., Ltd., Osaka, Japan) and an RT-PCR detection system (Bio-Rad Laboratories Inc.). Thermocycling parameters were: initial denaturation for 1 min at 95 °C; followed by 40 denaturation cycles at 95 °C for 15 s, annealing for 15 s at 60 °C, and elongation at 72 °C for 60 s. Samples were run in triplicate. Relative gene expression was analyzed with reference to GAPDH expression and the 2^-△△Ct^ method: ①Normalize target genes using internal reference genes:

$$\mathrm{\Delta CT}\;\left(\text{treatment group}\right)=\mathrm{CT}\;\left(\text{target gene}\right)-\mathrm{CT}\;\left(\text{internal reference gene}\right);\mathrm{\Delta CT}\;\left(\text{blank group}\right)=\mathrm{CT}\;\left(\text{target gene}\right)-\mathrm{CT}\;\left(\text{reference gene}\right)$$

②Normalize the ΔCT value of the treatment group using the ΔCT value of the blank control:

$$\text{ΔΔCT}=\mathrm{\Delta CT}\;\left(\text{treatment group}\right)-\mathrm{\Delta CT}\;\left(\text{blank group}\right)$$

③Calculate the expression level ratio:

2−ΔΔCT=relative expression level of the target gene

### Western blotting

Samples were homogenized and total protein extracted using radioimmunoprecipitation assay buffer (Beyotime Institute of Biotechnology, Haimen, China). Protein concentrations were determined using a bicinchoninic acid assay kit (Beyotime Institute of Biotechnology, Haimen, China), after which equal protein quantities were loaded onto sodium dodecyl sulfate polyacrylamide gels (10%) (Aspen, Wuhan, China). After electrophoresis, proteins were transferred to nitrocellulose membranes (Pall Corp., New York, NY, USA) and blocked in nonfat dry milk (5%) for 2 h at room temperature. Membranes were incubated at 4 °C overnight with primary antibodies against VEGF-A (1:1,000; ab46154; Abcam, Cambridge, UK), PDGF-BB (1:1,000; ab16829; Abcam), TGF-β1 (1:500; ab64715; Abcam), EGF (1:500; ab77851; Abcam), SDF-1 (1:500; ab18919; Abcam), and GAPDH (1:10,000; ab37168; Abcam). Finally, membranes were incubated with a secondary horseradish peroxidase-conjugated antibody (Aspen Wuhan, China) for 1 h, and protein bands detected using an enhanced chemiluminescence substrate (Beyotime Institute of Biotechnology, Haimen, China).

### Statistical analysis

Data were presented as the mean ± standard deviation. Statistical significance was assessed by one-way analysis of variance. Statistical analyses were performed using SPSS 18.0 software (SPSS, Chicago, IL, USA). Differences between groups were considered statistically significant at *P* < 0.05 or *P* < 0.01.

## Results

### Basic characteristics of the animal model

To confirm the stability of the diabetic model, we monitored the blood glucose and body weight of rats in each group before modeling (baseline) and at the experimental endpoint (day 7). As shown in Table 1, all rats maintained a stable hyperglycemic state (blood glucose >16.7 mmol/L) throughout the experimental period. Furthermore, there were no significant differences in blood glucose or body weight between the NPWT and control groups at baseline, indicating balanced group allocation. By day 7, the body weight of the control group was significantly lower than that of the NPWT group (*p* < 0.05), suggesting that NPWT may have a positive impact on the overall systemic condition of the animals.

**Table 1:** Basic characteristics of the animal model.

Group	Time point	n	NPWT	Control	*P* value
Blood Glucose (mmol/L)	Baseline (Day 0)	15	20.8 ± 2.5	20.1 ± 2.8	0.462
	Endpoint (Day 7)	15	21.1 ± 2.9	21.0 ± 3.1	0.934
Body Weight (g)	Baseline (Day 0)	15	245.3 ± 8.2	243.9 ± 7.5	0.631
	Endpoint (Day 7)	15	248.1 ± 9.7	239.4 ± 10.5	0.022

Data are presented as mean ± standard deviation; p < 0.05 was considered statistically significant.

### Altered VEGF, PDGF-BB, EGF, TGF, and SDF-1 expression

**Fig. 1:**
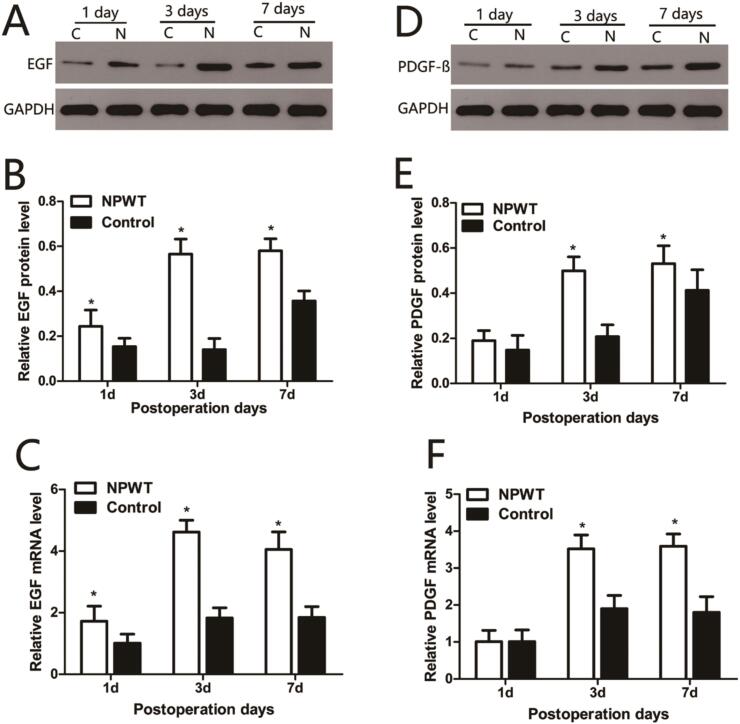
Expression of EGF, PDGF-BB in the wound during wound healing. (A) Representative Western blots showing the protein levels of EGF in both groups. (B) Statistical analysis protein levels of EGF in both groups. (C) EGF gene expression levels were assessed by qRT-PCR in the both groups. (D) Representative Western blots showing the protein levels of PDGF-BB in both groups. (E) Statistical analysis protein levels of PDGF-BB in both groups. (F) PDGF-BB gene expression levels were assessed by qRT-PCR in the both groups, *P < 0.05.

Western blots were used to quantitatively analyze PDFG-BB, TGF-β1, EGF, VEGF-A, and SDF-1 protein expression. EGF expression (Fig. 1A) gradually increased in the experimental group from day 1 to day 7 and levels were significantly higher when compared with the control group during this period (*P* < 0.0*5*) (Fig. 1B). EGF mRNA expression trends were consistent with protein expression profiles in both groups. Statistical analyses are shown (Fig. 1C).

PDGF-BB protein expression (Fig. 1D) gradually increased in the experimental group from day 1 to 7, peaked on day 7, and was significantly higher when compared with the control group over this time (*P* < 0.05). PDGF-BB mRNA expression levels (Fig. 1E) were consistent with protein expression profiles in both groups. Statistical analyses are shown (Fig. 1F).

**Fig. 2:**
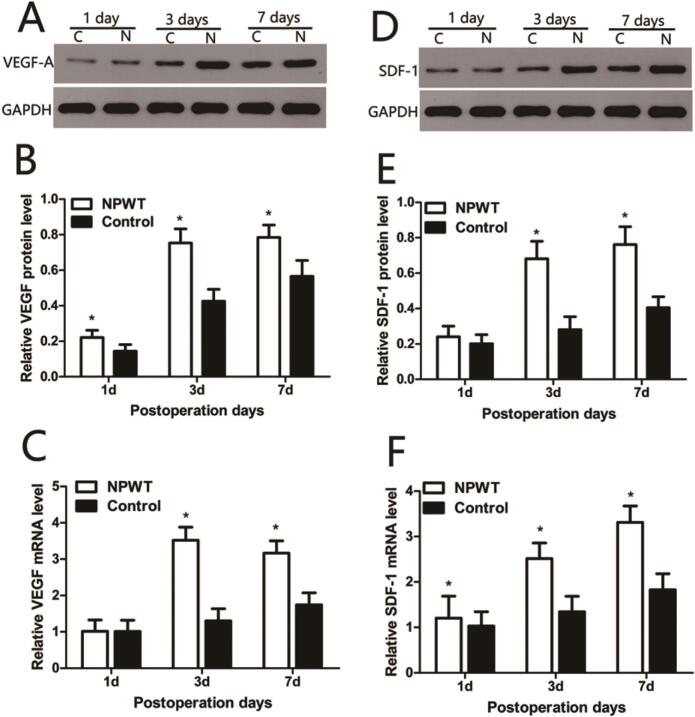
Expression of VEGF-A, SDF-1 in the wound during wound healing. (A) Representative Western blots showing the protein levels of VEGF-A in both groups. (B) Statistical analysis protein levels of VEGF in both groups. (C) VEGF gene expression levels were assessed by qRT-PCR in the both groups. (D) Representative Western blots showing the protein levels of SDF-1 in both groups. (E) Statistical analysis protein levels of SDF-1 in both groups. (F) SDF-1 gene expression levels were assessed by qRT-PCR in the both groups, *P < 0.05.

VEGF-A protein expression (Fig. 2A) gradually increased from day 1 to 7, however it was significantly increased when compared with the control group over this time (*P* < 0.05) (Fig. 2B). VEGF-A mRNA expression levels were consistent with protein expression patterns in both groups. Statistical analyses are shown (Fig. 2C).

SDF-1 protein expression (Fig. 2D) gradually increased in both groups on days 1, 3, and 7 and was significantly higher in the experimental groups when compared with the control group over this period (*P* < 0.0*5*) (Fig. 2E). SDF-1 mRNA expression levels (Fig. 2F) were consistent with protein expression profiles in both groups, and were significantly higher in the experimental group when compared with control group during wound healing (*P* < 0.05).

**Fig. 3:**
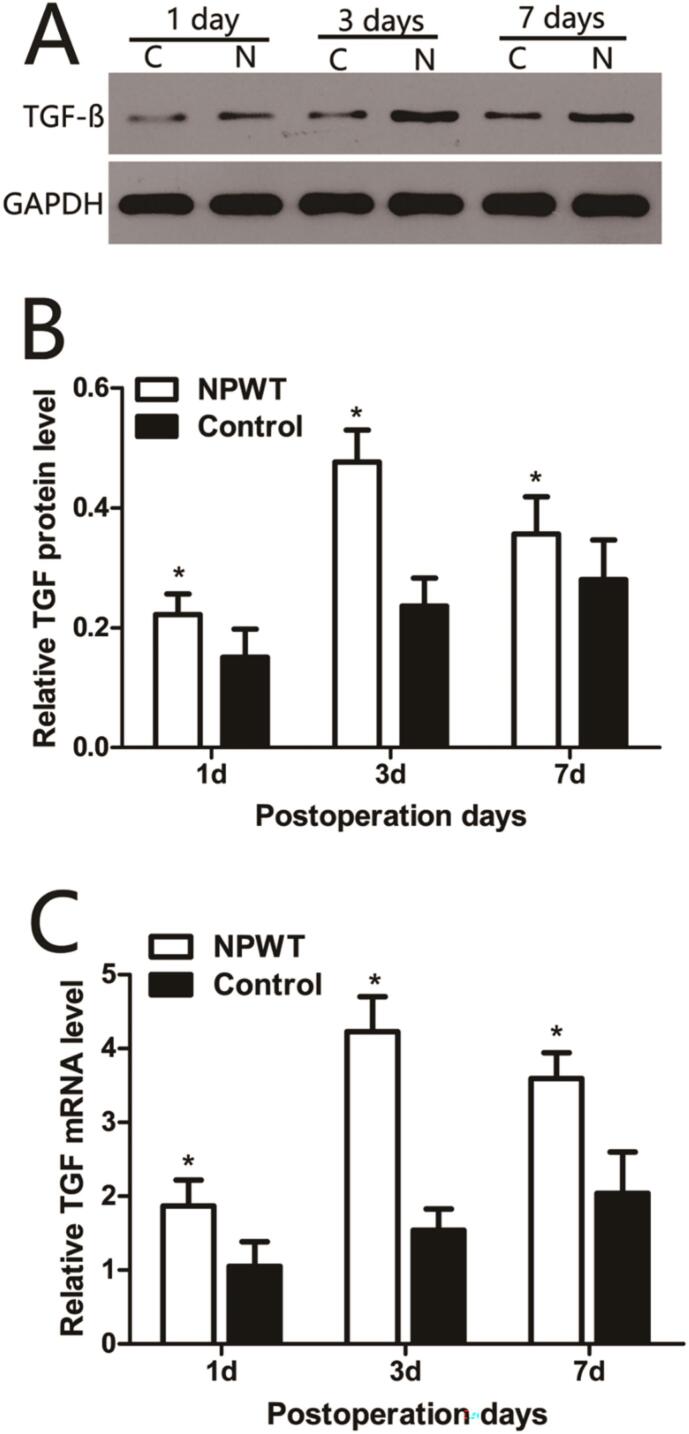
Expression of TGF-ß in the wound during wound healing. (A) Representative Western blots showing the protein levels of TGF-ß in both groups. (B) Statistical analysis protein levels of TGF-ß in both groups. (C) TGF-ß gene expression levels were assessed by qRT-PCR in the both groups, *P < 0.05.

TGF-β1 protein expression (Fig. 3A) gradually increased in both groups from day 1 to 7, however, it was significantly higher in the experimental group when compared with the control group on days 1, 3, and 7 (P < 0.05) (Fig. 3B). TGF-β1 mRNA expression levels (Fig. 3C) were consistent with protein expression profiles in both groups, and were significantly higher in the experimental group when compared with the control group across all time points (*P* < 0.05).

### Flow cytometry

**Fig. 4:**
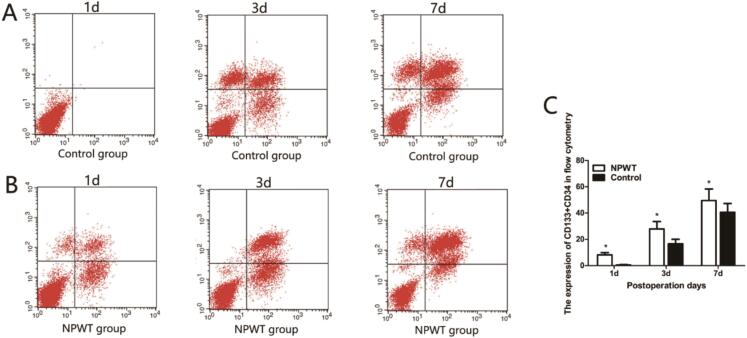
Flow cytometry was used to detect CD133 + CD34. (A) Flow cytometry was used to detect CD133 + CD34 in control group during the wound healing. (B) Flow cytometry was used to detect CD133 + CD34 in experiment group during the wound healing. (C) Statistical analysis experiment levels of CD133 + CD34 in both groups, *P < 0.05.

Flow cytometry was used to quantitate endothelial progenitor cells (EPCs), bone mesenchymal stem cells (MSCs), and circulating fibroblasts. CD34 and CD133 were used to label EPCs (Fig. 4A, B). EPC expression was gradually increased in both groups at 1, 3, and 7 days, with higher statistically significantly differences in the control group when compared with the experimental group (Fig. 4C).

**Fig. 5:**
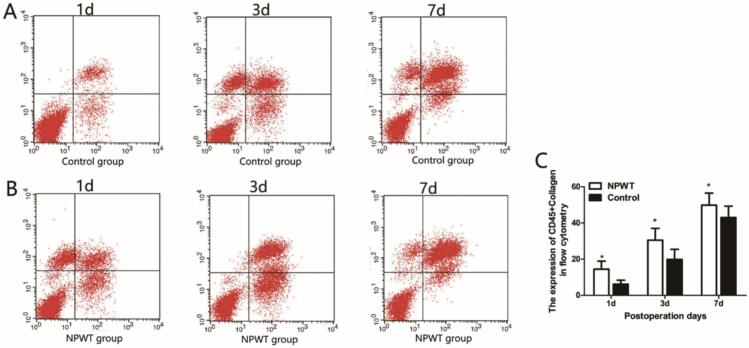
Flow cytometry was used to detect CD45 + Collagen. (A) Flow cytometry was used to detect CD45 + Collagen in control group during the wound healing. (B) Flow cytometry was used to detect CD45 + Collagen in experiment group during the wound healing. (C) Statistical analysis experiment levels of CD45 + Collagen in both groups, *P < 0.05.

Bone MSCs have major roles in wound healing^7^. This study used CD29 and CD44 to label MSCs, and the results showed that the expression levels of CD29 and CD44 gradually increased (Fig. 5A and B). Expression in the experimental group was significantly higher when compared with the control group (*P* < 0.0*5*) (Fig. 5C).

**Fig. 6:**
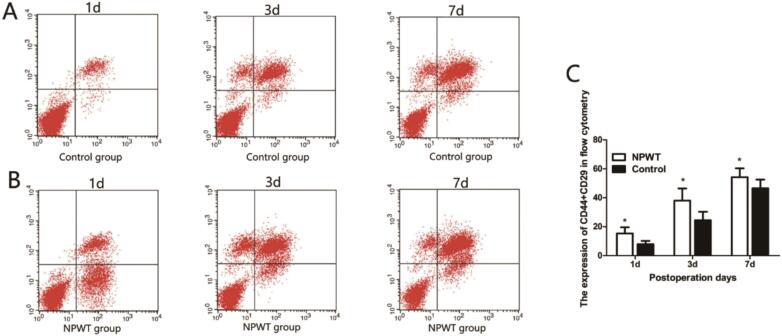
Flow cytometry was used to detect CD44 + CD29. (A) Flow cytometry was used to detect CD44 + CD29 in control group during the wound healing. (B) Flow cytometry was used to detect CD44 + CD29 in experiment group during the wound healing. (C) Statistical analysis experiment levels of CD44 + CD29 in both groups, *P < 0.05.

Circulating fibroblasts are a subgroup of leukocytes with fibroblast characteristics and mainly express CD45 or collagen 1 [9], [10]. As shown (Fig. 6A, B), fibroblast expression gradually increased on days 1, 3, and 7, peaked on day 7, with levels in the experimental group significantly higher when compared with the control group (P < 0.0*5*) (Fig. 6C).

### Wound changes

The macroscopic progression of wound healing was documented through periodic photographic imaging (Fig. 8). Dynamic curve of the healing rate (Fig. 8). As shown, on postoperative days 3 and 7, the extent of wound contraction in the NPWT group was markedly superior to that in the control group.

## Discussion

Wound healing is a highly coordinated and sequentially overlapping angiogenic process, but based on the tissue response after injury, it can be divided into four distinct phases: 1) hemostasis, 2) inflammation, 3) proliferation, and 4) remodeling (maturation) [11]. Each phase represents a complex wound microenvironment composed of different growth factors, cell types, and cytokines that coordinate cell migration, proliferation, extracellular matrix deposition, and neovascularization and maturation [12]. Neovascularization is an integral process throughout all stages of wound healing; it provides nutrients to the cells regulating the wound healing process [13] and requires complex interactions among multiple growth factors, cytokines, and chemokines. New blood vessel formation occurs primarily through angiogenesis (and vasculogenesis), involving endothelial cells and pericytes, where new capillaries sprout from existing vessels. When the vasculature is compromised by wounding, endothelial cells migrate and proliferate to form tubular network structures that eventually develop into new blood vessels [14], [15], [16]. Vasculogenesis describes the de novo formation of new blood vessels during embryogenesis, where precursor cells migrate and differentiate into endothelial cells that coalesce into vascular plexuses and a primordial vasculature. Crucially, both angiogenesis and vasculogenesis are regulated by growth factors, cytokines, chemokines, and precursor cells.

Although the positive effects of the NPWT approach are recognized, animal experimental studies and clinical trials have not yet thoroughly investigated these phenomena from the perspective of analyzing NPWT fluid composition, demonstrating how the fluid may benefit/hinder wound healing, and studying growth factor expression and cells in wound healing fluid. This study collected and analyzed the body fluids/exudates produced by negative pressure wound therapy or gauze dressings. The results showed that during the wound healing process, the expression levels of vascular endothelial growth factor A, transforming growth factor β, epidermal growth factor, platelet-derived growth factor BB, and stromal cell-derived factor 1 gradually increased in the experimental group.

**Fig. 7:**
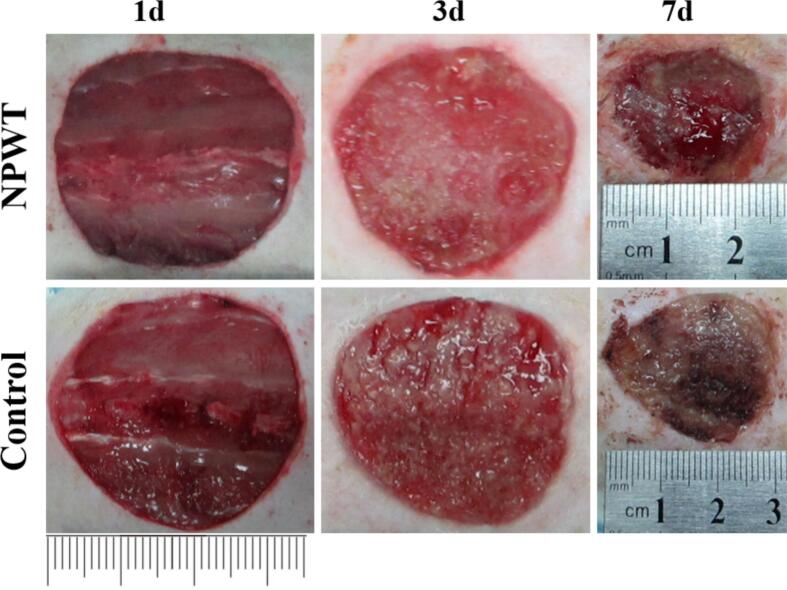
As shown, on postoperative days 3 and 7, the extent of wound contraction in the NPWT group was markedly superior to that in the control group.

The core finding of this study is that in a diabetic rat wound model, compared to the control group, the NPWT drainage fluid showed a significant and sustained increase in both protein and mRNA levels of key growth factors/chemokines such as VEGF-A, PDGF-BB, TGF-β1, EGF, and SDF-1, along with a significant increase in the numbers of circulating endothelial progenitor cells, mesenchymal stem cells, and fibroblasts. These findings, combined with our newly added wound area change graph (Fig. 7, showing faster healing in the NPWT group), provide new molecular and cellular-level evidence for understanding how NPWT optimizes the wound microenvironment.

**Fig. 8:**
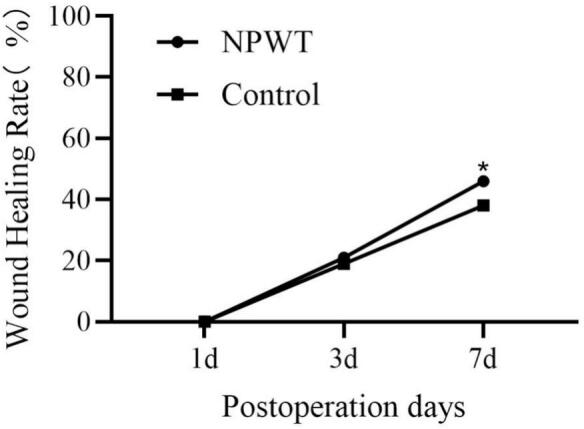
Dynamic curve of the healing rate.

The traditional view holds that NPWT primarily promotes healing through macroscopic mechanical forces (e.g., reducing edema, promoting granulation tissue growth) [17]. This study suggests that NPWT may not only remove excess exudate and some inflammatory mediators locally but, more importantly, the specific pro-healing factors (e.g., VEGF-A, SDF-1) and stem cells enriched in its drainage fluid dynamically reflect an active shift of the wound microenvironment from an inflammatory state towards a proliferative and reparative state under NPWT intervention. Specifically, the high levels of pro-angiogenic factors like VEGF-A and SDF-1 in the drainage fluid, together with the enrichment of reparative cells like EPCs and MSCs, collectively point towards a microenvironment “remodeled” by NPWT that is more inclined towards regeneration rather than sustained inflammation. This does not simply mean these beneficial components are “wastefully” removed; it is more likely an extension of NPWT's “debridement effect” at the molecular and cellular level, breaking the common “inflammatory stagnation” state in diabetic wounds and clearing obstacles for the subsequent proliferative phase.

Fibroblasts play a key role in granulation tissue formation during wound healing; they migrate from adjacent dermis into the wound area in response to chemokines, cytokines, and growth factors [18], [19]. Previous studies reported that circulating fibroblasts develop and differentiate in the early stages of wound healing, and this process is regulated by key growth factors, chemokines, and cytokines [20]. In our study, wound drainage gradually increased during the early wound healing stages after NPWT and showed a substantial number of circulating fibroblasts being removed by the negative pressure. Circulating fibroblasts also express various markers, but primarily CD34, CD45, collagen I, and CD133 [21], [22], [23]. This study used double labeling staining for CD34 and collagen I to identify circulating fibroblasts. The results showed that during the wound healing process, the number of fibroblasts in both groups gradually increased, but the cell count in the experimental group was significantly higher than in the control group (*P* < 0.05). Previous research reported high levels of circulating fibroblasts found in granulation tissue after NPWT [20]. Although negative pressure promotes fibroblast differentiation and proliferation [20], the number of circulating fibroblasts in the drainage fluid cannot be ignored. Therefore, negative pressure significantly promotes the proliferation of circulating fibroblasts, which is beneficial for wound healing.

The VEGF family plays a key role in angiogenesis, vascular remodeling, and neovessel stabilization during wound healing [24], [25], [26], [27], and its sustained high expression is closely associated with the synchronous increase in EPC numbers. This suggests that NPWT not only upregulates pro-angiogenic signaling molecules but may also promote the mobilization of a circulating cell pool capable of homing to the wound and participating in new blood vessel formation [28]. This provides a potential cellular explanation for the clinically observed phenomenon of NPWT improving wound blood perfusion.

PDGF-BB promotes the stability of capillary networks and induces pericyte recruitment and differentiation into the capillary wall, thereby stabilizing vessels and promoting capillary structural integrity [29], [30], [31]. PDGF-BB and EGF act synergistically to promote pericyte recruitment to the capillary wall, thus stabilizing vessels, while PDGF-BB and VEGF-A promote angiogenesis [32], [33], [34]. PDGF-BB and TGF-β1 are potent mitogens and chemoattractants, crucial for the proliferation and migration of fibroblasts and smooth muscle cells, as well as extracellular matrix synthesis. Their high expression in this study, coinciding with the increased number of circulating fibroblasts, strongly suggests that NPWT, by enhancing the supply and activity of these cells, lays the material foundation for granulation tissue filling and the subsequent remodeling phase.

SDF is a member of the CXC family and primarily acts through the CXCR4 receptor. SDF-1 is a structural protein, and its expression is upregulated by extracellular matrix changes, inflammatory responses, mechanical force stimulation, and hypoxia [35], [36]. SDF-1 plays a key role in the inflammatory response; it primarily recruits lymphocytes to the wound and promotes angiogenesis during wound healing [37]. Its upregulated expression, coupled with the increased number of MSCs in the drainage fluid, suggests that NPWT may enhance the recruitment of MSCs—with multidifferentiation potential and self-renewal capacity—to the wound by modulating the SDF-1/CXCR4 axis. These MSCs and the paracrine factors they secrete play a central role in modulating immunity, suppressing excessive inflammation, and promoting tissue regeneration.

The primary innovation of this study lies in extending the research perspective on NPWT from the wound bed itself to its drainage fluid. The systematic analysis of the drainage fluid makes it a “window of information” dynamically reflecting internal molecular and cellular events within the wound. Our results indicate that the levels of specific pro-healing components in the drainage fluid may be positively correlated with the wound's healing status. This provides preliminary theoretical basis for the future development of auxiliary tools based on biomarkers in drainage fluid for monitoring NPWT efficacy or predicting healing progression.

### Limitations and future directions

This study has certain limitations. First, the findings from the animal experiments require validation in clinical patient samples. Second, although we observed associations between molecular/cellular changes and macroscopic healing, we have not directly demonstrated that the alterations in the composition of the drainage fluid are the direct cause of the improved healing. Future research could employ techniques such as in vivo tracing, gene knockout, or specific inhibitor blockade to further verify the causal role of specific factors (e.g., SDF-1) in NPWT-promoted healing. Additionally, including a “sham suction” control group (using a closed dressing without negative pressure) would help to more precisely isolate the effects attributable to the negative pressure itself.

## CRediT authorship contribution statement

**Muhaimaiti Abudurezhake:** Writing – original draft, Formal analysis. **Yifei Huang:** Writing – review & editing, Supervision. **Hailong Wang:** Formal analysis. **Gulinuer Aili:** Resources. **Yamei Xu:** Resources, Formal analysis. **Yajun Tian:** Resources, Formal analysis. **Zhanjun Ma:** Writing – original draft, Supervision, Conceptualization.

## Ethical approval

Ethics committee approval for The negative effect of negative pressure wound treatment–from the perspective of drainage fluid composition was obtained from the Ethics Committee of the Zhongnan Hospital of Wuhan University (Wuhan, China) (2021006). All animal experiments in this study complied with the ARRIVE guidelines and were conducted in accordance with the U.K. Animals (Scientific Procedures) Act 1986 and associated ethical guidelines.

## Funding

This study was supported by Natural Science Foundation of Xinjiang Uygur Autonomous Region (NO. 2023D01C144), the Key R&D Plan Project of Xinjiang Uygur Autonomous Region (NO. 2022B03011-1), Xinjiang Uygur Autonomous Region Science and Technology Innovation Leading Talent Project - High Level Leading Talent Project (No. 2022TSYCLJ0027) and Science and technology prosperity AKESU project (00121) and (00417).

## Declaration of competing interest

The authors declare that they have no known competing financial interests or personal relationships that could have appeared to influence the work reported in this paper.

## References

[1] N G., S C., El G, M Em, R A., C L. (2022). Negative pressure wound therapy for surgical wounds healing by primary closure. Cochrane Database Syst Rev.

[2] Cocjin H.G.B., Jingco J.K.P., Tumaneng F.D.C., Coruña J.M.R. (2019). Wound-healing following negative-pressure wound therapy with use of a locally developed AquaVac system as compared with the vacuum-assisted closure (VAC) system. J Bone Joint Surg Am.

[3] Guoqi W., Zhirui L., Song W., Tongtong L., Lihai Z., Licheng Z. (2018). Negative pressure wound therapy reduces the motility of pseudomonas aeruginosa and enhances wound healing in a rabbit ear biofilm infection model. Antonie Van Leeuwenhoek.

[4] Ma Z., Li Z., Shou K., Jian C., Li P., Niu Y. (2017). Negative pressure wound therapy: regulating blood flow perfusion and microvessel maturation through microvascular pericytes. Int J Mol Med.

[5] Ma Z., Shou K., Li Z., Jian C., Qi B., Yu A. (2016). Negative pressure wound therapy promotes vessel destabilization and maturation at various stages of wound healing and thus influences wound prognosis. Exp Ther Med.

[6] Han C., Singla R.K., Wang C. (2025). Application of biomaterials in diabetic wound healing: the recent advances and pathological aspects. Pharmaceutics.

[7] Lohr J.M., Raffetto J.D., Dexter D.J., Regulski M.J., Edmonds M.E., Ozsvath K.J. (2025). A synergistic multimodality treatment approach to address the key drivers of wound chronicity. J Vasc Surg Venous Lymphat Disord.

[8] Chen L., Zhang S., Da J., Wu W., Ma F., Tang C. (2021). A systematic review and meta-analysis of efficacy and safety of negative pressure wound therapy in the treatment of diabetic foot ulcer. Ann Palliat Med.

[9] Gilpin S.E., Lung K.C., Sato M., Singer L.G., Keshavjee S., Waddell T.K. (2012). Altered progenitor cell and cytokine profiles in bronchiolitis obliterans syndrome. J Heart Lung Transplant.

[10] LaPar D.J., Burdick M.D., Emaminia A., Harris D.A., Strieter B.A., Liu L. (2011). Circulating fibrocytes correlate with bronchiolitis obliterans syndrome development after lung transplantation: a novel clinical biomarker. Ann Thorac Surg.

[11] Reinke J.M., Sorg H. (2012). Wound repair and regeneration. Eur Surg Res, Eur Chir Forsch, Rech Chir Eur.

[12] Ko S.H., Nauta A., Wong V., Glotzbach J., Gurtner G.C., Longaker M.T. (2011). The role of stem cells in cutaneous wound healing: what do we really know?. Springer Spec Surg S.

[13] King A., Balaji S., Keswani S.G., Crombleholme T.M. (2014). The role of stem cells in wound angiogenesis. Adv Wound Care.

[14] Folkman J. (2003). Fundamental concepts of the angiogenic process. Curr Mol Med.

[15] Sato Y. (2012). The vasohibin family: novel regulators of angiogenesis. Vascul Pharmacol.

[16] Raza A., Franklin M.J., Dudek A.Z. (2010). Pericytes and vessel maturation during tumor angiogenesis and metastasis. Am J Hematol.

[17] Hasan M.Y., Teo R., Nather A. (2015). Negative-pressure wound therapy for management of diabetic foot wounds: A review of the mechanism of action, clinical applications, and recent developments. Diabet. Foot Ankle.

[18] Schultz G.S., Wysocki A. (2009). Interactions between extracellular matrix and growth factors in wound healing. Wound Repair Regen.

[19] Hinz B., Phan S.H., Thannickal V.J., Galli A., Bochaton-Piallat M.-L., Gabbiani G. (2007). The myofibroblast: one function, multiple origins. Am J Pathol.

[20] Chen D., Zhao Y., Li Z., Shou K., Zheng X., Li P. (2017). Circulating fibrocyte mobilization in negative pressure wound therapy. J Cell Mol Med.

[21] Circulating fibrocytes define a new leukocyte subpopulation that mediates tissue repair - PubMed n.d. https://pubmed.ncbi.nlm.nih.gov/8790603/ (accessed December 11, 2025). PMC2229929 8790603

[22] Chesney J., Metz C., Stavitsky A.B., Bacher M., Bucala R. (1998). Regulated production of type I collagen and inflammatory cytokines by peripheral blood fibrocytes. J Immunol (Baltim Md,: 1950).

[23] Fibrocytes in the tumor microenvironment - PubMed n.d. https://pubmed.ncbi.nlm.nih.gov/32036606/ (accessed December 11, 2025).

[24] Isner J.M., Pieczek A., Schainfeld R., Blair R., Haley L., Asahara T. (1996). Clinical evidence of angiogenesis after arterial gene transfer of phVEGF165 in patient with ischaemic limb. Lancet (Lond Engl).

[25] Baumgartner I., Pieczek A., Manor O., Blair R., Kearney M., Walsh K. (1998). Constitutive expression of phVEGF165 after intramuscular gene transfer promotes collateral vessel development in patients with critical limb ischemia. Circulation.

[26] Ap V., H K., S A., S Ad, B Ab (2019). Therapeutic strategies for enhancing angiogenesis in wound healing. Adv Drug Deliv Rev.

[27] Odent Grigorescu G., Rosca A.-M., Preda M.B., Tutuianu R., Simionescu M., Burlacu A. (2017). Synergic effects of VEGF-a and SDF-1 on the angiogenic properties of endothelial progenitor cells. J Tissue Eng Regen Med.

[28] Hasan M.Y., Teo R., Nather A. (2015). Negative-pressure wound therapy for management of diabetic foot wounds: a review of the mechanism of action, clinical applications, and recent developments. Diabet Foot Ankle.

[29] Ma Z., Li Z., Shou K., Jian C., Li P., Niu Y. (2017). Negative pressure wound therapy: regulating blood flow perfusion and microvessel maturation through microvascular pericytes. Int J Mol Med.

[30] Ma Z., Shou K., Li Z., Jian C., Qi B., Yu A. (2016). Negative pressure wound therapy promotes vessel destabilization and maturation at various stages of wound healing and thus influences wound prognosis. Exp Ther Med.

[31] Hellberg C., Ostman A., Heldin C.-H. (2010). PDGF and vessel maturation. Recent Results Cancer Res, Fortschr Krebsforsch. Prog Dans Rech Sur Cancer.

[32] The role of pericytes in blood-vessel formation and maintenance - PubMed n.d. https://pubmed.ncbi.nlm.nih.gov/16212810/ (accessed December 11, 2025). 10.1215/S1152851705000232 PMC1871727 16212810

[33] Reyahi A., Nik A.M., Ghiami M., Gritli-Linde A., Pontén F., Johansson B.R. (2015). Foxf2 is required for brain pericyte differentiation and development and maintenance of the blood-brain barrier. Dev Cell.

[34] Endothelial-derived PDGF-BB and HB-EGF coordinately regulate pericyte recruitment during vasculogenic tube assembly and stabilization - PubMed n.d. https://pubmed.ncbi.nlm.nih.gov/20739660/ (accessed December 11, 2025). 10.1182/blood-2010-05-286872 PMC2996127 20739660

[35] Glass G.E., Murphy G.F., Esmaeili A., Lai L.-M., Nanchahal J. (2014). Systematic review of molecular mechanism of action of negative-pressure wound therapy. Br J Surg.

[36] Progenitor cell trafficking is regulated by hypoxic gradients through HIF-1 induction of SDF-1 - PubMed n.d. https://pubmed.ncbi.nlm.nih.gov/15235597/ (accessed December 11, 2025). 10.1038/nm1075 15235597

[37] Toksoy A., Müller V., Gillitzer R., Goebeler M. (2007). Biphasic expression of stromal cell-derived factor-1 during human wound healing. Br J Dermatol.

